# Fecal carriage, transferable β-lactam resistance, and efflux pump contribution in third-generation cephalosporin-resistant *Escherichia coli* from small-scale farm animals and workers in northern Thailand

**DOI:** 10.14202/vetworld.2026.366-379

**Published:** 2026-01-30

**Authors:** Uttapoln Tansawai, Pannika R. Niumsup

**Affiliations:** 1 Department of Science, Faculty of Science and Agricultural Technology, Rajamangala University of Technology Lanna Phitsanulok, Phitsanulok, 65000, Thailand; 2 Department of Microbiology and Parasitology, Faculty of Medical Science, Naresuan University, Phitsanulok, 65000, Thailand

**Keywords:** antimicrobial resistance, efflux pump, *Escherichia coli*, extended-spectrum beta-lactamase, multidrug resistance, One Health, plasmid-mediated resistance, small-scale farms, Thailand, third-generation cephalosporin resistance, zoonotic transmission

## Abstract

**Background and Aim:**

Food-producing animals are recognized reservoirs of antimicrobial-resistant bacteria with zoonotic potential. Third-generation cephalosporin-resistant (3GC-R) *Escherichia coli* is of particular public health concern due to its association with extended-spectrum β-lactamase (ESBL) and plasmid-mediated AmpC enzymes. This study aimed to determine the prevalence, resistance mechanisms, transferability of resistance genes, efflux pump contribution, and genetic relatedness of 3GC-R *E. coli* isolated from farm animals and workers on a small-scale farm in lower northern Thailand.

**Materials and Methods:**

A total of 265 fecal samples were collected from laying hens (n = 210), cattle (n = 33), swine (n = 19), and farm workers (n = 3). Isolation of 3GC-R *E. coli* was performed using cefotaxime-supplemented selective media. Antimicrobial susceptibility was determined by disk diffusion and minimum inhibitory concentration (MIC) assays. The presence of *bla*_CTX-M_ and *bla*_CMY-2_ genes was identified by polymerase chain reaction and sequencing. Conjugation assays and plasmid replicon typing assessed gene transferability. Efflux pump involvement in ceftazidime resistance was evaluated using phenylalanine-arginine β-naphthylamide. Genetic relatedness was analyzed by pulsed-field gel electrophoresis.

**Results:**

Overall, 15.8% of samples yielded 3GC-R *E. coli*, with the highest prevalence observed in swine (47.4%), followed by cattle (27.3%) and laying hens (10.5%). Multidrug resistance was detected in 57.1% of isolates. The majority carried *bla*_CTX-M_ alone (69.0%) or in combination with *bla*_CMY-2_ (21.4%), and both genes were transferable via IncF and IncI1-I plasmids. A ≥4-fold reduction in ceftazidime MICs in the presence of an efflux pump inhibitor was observed in 38.7% of isolates. While genetically identical strains were detected among different animal species, no clonal transmission between animals and workers was identified.

**Conclusion:**

Small-scale farms in Thailand harbor 3GC-R *E. coli* with transferable resistance determinants and multiple resistance mechanisms, underscoring their role in antimicrobial resistance dissemination. These findings highlight the need for strengthened biosecurity, antimicrobial stewardship, and integrated One Health surveillance in rural farming systems.

## INTRODUCTION

Diseases caused by antibiotic-resistant bacteria (ARB) are responsible for substantial global mortality and economic losses each year. Data from the World Health Organization (WHO) Global Antimicrobial Resistance and Use Surveillance System (GLASS), derived from more than 23 million bacterial infections across 104 countries, indicate widespread resistance to essential antibiotics, particularly among Gram-negative bacteria. The highest resistance burden has been reported in Southeast Asia (31.1%), exceeding the global median of 17.2% [1]. Furthermore, the Global Burden of Disease 2021 Antimicrobial Resistance (AMR) Collaborators estimated that 1.14 million deaths were attributable to ARB worldwide between 1990 and 2021, spanning 204 countries [2]. Notably, mortality associated with ARB infections has increased by more than 80% in recent years, particularly among individuals aged ≥70 years [2].

The emergence and dissemination of ARB are largely driven by the excessive and inappropriate use of antibiotics in both human medicine and livestock production systems. A recent study documented the widespread use of critically important antimicrobials in food-producing animals across Southeast Asia, including Thailand [3]. Although initiatives promoting rational antibiotic use are increasingly being implemented, ARB continue to be detected in humans, animals, and the environment. Consequently, a One Health approach is essential to address ARB, as resistant bacteria and resistance determinants can rapidly disseminate across human–animal–environment interfaces.

*Escherichia coli* is a commensal bacterium of the intestinal tracts of humans and animals and is widely distributed in agricultural and environmental settings. Certain *E. coli* strains are important pathogens, causing both intestinal and extraintestinal infections, including uncomplicated cystitis, pneumonia, and bacteremia. As one of the most frequently identified antibiotic-resistant organisms, *E. coli* contributes significantly to morbidity and mortality worldwide. Its capacity to acquire resistance through horizontal gene transfer and chromosomal mutations further exacerbates this public health challenge [4]. In 2024, the WHO classified third-generation cephalosporin-resistant (3GC-R) *E. coli* within the “critical group” of bacterial pathogens requiring urgent research, development, and public health interventions [5]. According to GLASS, 3GC-R *E. coli* ranked sixth among the top ten bacterial pathogens exhibiting increasing AMR trends [1]. Clinical evidence indicates that bloodstream infections caused by 3GC-R *E. coli* are associated with significantly higher mortality compared with infections caused by cephalosporin-susceptible strains [6]. In Thailand, surveillance data from 111 hospitals identified 3GC-R *E. coli* as the first- and third-leading causes of bloodstream infections in community and hospital settings, respectively [7]. Moreover, numerous studies have documented resistance to third-generation cephalosporins, including cefotaxime, ceftriaxone, and ceftazidime, in *E. coli* isolates from diverse sources, particularly food-producing animals [3, 4].

Resistance to third-generation cephalosporins in *E. coli* is primarily mediated by the production of extended-spectrum β-lactamases (ESBLs), especially CTX-M enzymes, and plasmid-mediated AmpC β-lactamases (pAmpC) [4]. Genes encoding ESBLs and pAmpC are frequently located on mobile plasmids that often co-harbor resistance determinants to other antimicrobial classes, such as fluoroquinolones and aminoglycosides, thereby promoting multidrug resistance (MDR). A recent global analysis of 113,139 *E. coli* isolates reported MDR and ESBL-producing prevalence rates of 26.6% and 18.8%, respectively [8]. In addition to β-lactamase production, resistance in Gram-negative bacteria is further enhanced by efflux pumps belonging to the resistance–nodulation–division (RND) superfamily, which actively expel a wide range of antibiotics and contribute to MDR phenotypes. Inhibition of these efflux systems has been proposed as a strategy to restore antibiotic efficacy and mitigate resistance [9]. Several efflux pump inhibitors (EPIs) have been identified, among which phenylalanine-arginine β-naphthylamide (PAβN) has demonstrated broad-spectrum activity against Gram-negative bacteria and has been shown to reduce minimum inhibitory concentrations (MICs) of multiple antibiotics in Enterobacterales and *Pseudomonas aeruginosa* [10–12].

The rapid spread of 3GC-R *E. coli* among humans, animals, and environmental reservoirs represents a growing therapeutic and public health concern. In Thailand, data from the National AMR Surveillance Center in 2024, encompassing 76,236 clinical *E. coli* isolates from 94 hospitals, revealed resistance rates of 44.0% to cefotaxime, 29.8% to ceftazidime, and 42.4% to ceftriaxone [13]. Beyond clinical settings, 3GC-R *E. coli* has also been isolated from farm animals and meat products across multiple regions of Thailand, underscoring the importance of integrated surveillance and control strategies across the food production continuum [14–16].

Despite the growing body of evidence documenting the prevalence of 3GC-R *E. coli* in humans and food-producing animals in Thailand, important knowledge gaps remain. Most available studies have focused on single host species, commercial production systems, or either human or animal sectors in isolation, limiting understanding of resistance dynamics within shared farm ecosystems. Data from small-scale, family-owned farms, where animals of different species are raised in close proximity to humans and where biosecurity practices are often inconsistently applied, remain particularly scarce, especially in lower northern Thailand. Moreover, while the presence of ESBL- and plasmid-mediated AmpC-producing *E. coli* has been reported, few studies have simultaneously characterized phenotypic resistance profiles, specific β-lactamase genes, plasmid transferability, and clonal relatedness across multiple livestock species and farm workers within a single setting.

In addition, although efflux pump overexpression is recognized as an important complementary mechanism contributing to MDR in Gram-negative bacteria, its role in ceftazidime resistance among 3GC-R *E. coli* from livestock-associated sources in Thailand has been insufficiently investigated. Most surveillance studies rely solely on β-lactamase gene detection, potentially underestimating the contribution of non-enzymatic resistance mechanisms. Furthermore, information on the genetic relationships among 3GC-R *E. coli* isolates from different animal species and humans on small-scale farms remains limited, hindering the ability to infer potential transmission pathways and the relative contributions of clonal spread versus horizontal gene transfer. Addressing these gaps is essential to support evidence-based AMR surveillance and to inform One Health interventions tailored to smallholder farming systems.

The present study aimed to comprehensively investigate the occurrence, resistance characteristics, and dissemination potential of 3GC-R *E. coli* on a small-scale, multi-species farm in lower northern Thailand. Specifically, the study sought to determine the fecal carriage of 3GC-R *E. coli* among laying hens, swine, cattle, and farm workers; to characterize their antimicrobial susceptibility profiles and MDR patterns; and to identify the presence of key β-lactamase genes, including *bla*_CTX-M_ and *bla*_CMY-2_. In addition, the transferability of these resistance determinants and their associated plasmid replicon types were evaluated to assess the potential for horizontal gene dissemination.

Furthermore, this study aimed to elucidate the contribution of efflux pump activity to ceftazidime resistance using an EPI–based approach and to assess the genetic relatedness of 3GC-R *E. coli* isolates across host species using pulsed-field gel electrophoresis (PEGE). By integrating phenotypic, genotypic, and molecular epidemiological analyses within a One Health framework, this study sought to generate baseline data on AMR dynamics in small-scale livestock farming systems and to provide evidence supporting strengthened biosecurity measures and integrated AMR surveillance in rural Thailand.

## MATERIALS AND METHODS

### Ethical approval

This study involved fecal sampling of farm animals and farm workers. The farm owner provided verbal permission for the collection of fecal samples. Ethical approval was not required for animal sampling because only fecal samples were collected after defecation from the ground, without any direct contact with the animals.

The protocol for human sampling was approved by the Naresuan University Institutional Review Board (COA No. 83/2013), and written informed consent was obtained from all farm workers prior to their participation in the study.

All procedures involving 3GC-R *E. coli* were conducted in a biosafety level 2 laboratory and were performed in accordance with standard biosafety guidelines and institutional regulations.

### Study period, location, and farm management

This study was conducted between March and April 2016 on a small-scale local farm in Phitsanulok province (16°49′N, 100°15′E), located approximately 377 km north of Bangkok, a region characterized by high livestock farming density. The farm, situated 30 km from the city center, raises three types of livestock, including laying hens, swine, and cattle, with approximate population sizes of 2,000, 100, and 80 animals, respectively.

Laying hens were reared in rows of battery cages, with three hens housed per cage. Swine were housed in small groups of four to five animals per pen with concrete flooring. Cattle were raised in a large outdoor pen with a soil surface. All animals were raised in close proximity to one another within a distance of approximately 200 m and were managed by three farm workers.

Standardized husbandry practices were applied to all animals throughout the rearing period. Commercial feed and groundwater were provided. Farm workers entered the animal areas twice daily to feed and monitor the animals. Biosecurity measures were not consistently or strictly applied; however, farm workers changed their boots before entering and after leaving the animal areas. Wastewater generated from the farm was collected in a sedimentation tank to remove large solids, after which the effluent was discharged into the environment without further treatment. Sludge and animal manure were directly applied as fertilizers.

### Study design, sample size, and sampling strategy

Sample size estimation for laying hens was performed using the single-population proportion formula, assuming an expected prevalence of 27% based on a previous study [15], a margin of error of 5%, and a 95% confidence interval (CI). Calculations were conducted using Epitools (https://epitools.ausvet.com.au/).

One fecal sample was collected from each swine pen, whereas fecal samples from cattle were randomly collected. The final sample size varied depending on the availability of fecal material at the time of collection. Three farm workers agreed to provide fecal samples. In total, 265 fecal samples were collected, comprising samples from laying hens (n = 210), cattle (n = 33), swine (n = 19), and farm workers (n = 3) (Table 1).

**Table 1:** Prevalence of 3GC-R, ESBL–producing *Escherichia coli* and distribution of β-lactamase genes in fecal samples collected from farm animals and farm workers on a small-scale farm in northern Thailand.

Fecal samples (n)	No. (%) of 3GC-R *E. coli*	95% CI	No. (%) of ESBL-producing *E. coli*	95% CI	*bla*_CTX-M /_ *bla*_CMY_					

					*bla*_CTX-M-15_	*bla*_CTX-M-55_	*bla*_CTX-M-14_	*bla*_CTX-M-15_ + *bla*_CMY-2_	*bla*_CTX-M-14_ *+ bla*_CMY-2_	*bla*_CMY-2_
Laying hens (210)	22 (10.5)	6.7–15.4	12 (54.5)	32.2–75.6	2	7	2	5	3	3
Cattle (33)	9 (27.3)	13.3–45.5	7 (77.8)	40.0–97.2		8			1	
Swine (19)	9 (47.4)	24.4–71.1	7 (77.8)	40.0–97.2		5	3			1
Farm workers (3(	2 (66.7)	9.4–99.2	2 (100)	NA	1	1				
Total )265)	42 (15.8)	11.7–20.8	28 (66.7)	50.5–80.4	3	21	5	5	4	4

3GC-R = Third-generation cephalosporin-resistant, ESBL = Extended-spectrum β-lactamase, CI = Confidence interval, NA = Not applicable.

### Sample collection and transport

Fresh fecal samples were randomly collected from animals using sterile spoons and sterile containers. For laying hens, voided feces were collected from beneath each cage, whereas fecal samples from swine and cattle were collected from the ground. The portion of feces that had not touched the ground was carefully selected and collected without disturbing the animals.

Fecal samples from the three farm workers were collected using sterile Amies swabs (Deltalab, Barcelona, Spain). All samples were transported to the laboratory on ice and processed within 6 h of collection.

### Isolation and identification of 3GC-R *E. coli* strains

A total of 265 fecal samples, including samples from laying hens (n = 210), cattle (n = 33), swine (n = 19), and farm workers (n = 3), were collected. For animal fecal samples, 1 g of fecal material was suspended in 9 mL of tryptic soy broth (Oxoid, Basingstoke, UK) and incubated overnight at 37°C. Following incubation, the enriched cultures were streaked onto eosin methylene blue (EMB) agar (Oxoid) supplemented with cefotaxime (2 µg/mL) (EMB-CTX). Fecal swabs obtained from farm workers were directly inoculated onto EMB-CTX agar. All inoculated plates were incubated at 37°C for 24 h. From each sample, one presumptive *E. coli* colony displaying a metallic green sheen was selected. Species identification was confirmed using the RapID™ ONE System (REMEL Inc., KS, USA).

### Antibiotic susceptibility testing

Antibiotic susceptibility testing was performed against 12 antimicrobial agents using the disk diffusion method in accordance with Clinical and Laboratory Standards Institute (CLSI) guidelines [17]. Freshly grown colonies were suspended in normal saline, and the turbidity was adjusted to 0.5 McFarland standard (1.5 × 10^8^CFU/mL). The bacterial suspension was spread evenly onto Mueller–Hinton agar (Oxoid, UK) using a sterile cotton swab. Antibiotic disks were placed on the agar surface, and plates were incubated at 37°C for 16–18 h.

Inhibition zones were measured manually using standardized rulers and interpreted as susceptible, intermediate, or resistant according to CLSI criteria [17]. Intermediate results were considered indicative of susceptibility. Isolates exhibiting resistance to at least three antimicrobial classes were classified as multidrug-resistant (MDR) [18].

The tested antibiotics included seven β-lactams; amoxicillin/clavulanate (20/10 µg), ceftazidime (30 µg), cefpodoxime (10 µg), cefotaxime (30 µg), cefepime (30 µg), aztreonam (30 µg), and imipenem (10 µg), and five non-β-lactams; chloramphenicol (30 µg), gentamicin (10 µg), ciprofloxacin (5 µg), doxycycline (30 µg), and trimethoprim–sulfamethoxazole (1.25/23.75 µg) (Oxoid). *E. coli* ATCC (American Type Culture Collection) 25922 was used as the quality control strain. All antibiotic disks were stored in sealed containers in the dark at 2°C–4°C prior to use, according to the manufacturer’s instructions.

MICs for transconjugants were determined using the broth microdilution method following CLSI guidelines [17]. Two-fold serial dilutions of each antibiotic were prepared in 200 µL of cation-adjusted Mueller–Hinton broth (Oxoid) in 96-well microtiter plates. Bacterial suspensions were inoculated into each well at a final concentration of 5 × 10^5^ CFU/mL. Plates were incubated at 37°C for 18–20 h. *E. coli* ATCC 25922 was included as a control. MICs were defined as the lowest concentration of an antibiotic that inhibited visible bacterial growth.

Phenotypic detection of ESBL production was performed using the combination disk method according to CLSI guidelines [17]. Cefotaxime (30 µg) and ceftazidime (30 µg), alone and in combination with clavulanic acid (30/10 µg), were used (Becton, Dickinson and Company, MD, USA). ESBL production was defined as an increase of ≥5 mm in the inhibition zone diameter for antibiotic–clavulanate disks compared with the corresponding antibiotic disks without clavulanic acid. *E. coli* ATCC 25922 and a known ESBL-producing *E. coli* strain were used as negative and positive controls, respectively [15].

### Detection of β-lactamase genes (*bla*_CTX-M_ and *bla*_CMY-2_)

Detection of genes encoding β-lactamases (*bla*_CTX-M_ and *bla*_CMY-2_) was performed by polymerase chain reaction (PCR) using previously published primers and conditions [19, 20]. The *bla*_CTX-M_ alleles were identified using group-specific primers for group 1 and group 9. Full-length amplification of *bla*_CMY-2_-like genes was performed using previously described primers.

Individual colonies were suspended in 300 µL of distilled water and used as PCR templates. Amplification reactions were performed in a total volume of 20 µL containing 1 µL of template DNA, 1× PCR buffer, 1.5 mM MgCl_2_, 0.2 mM dNTPs, 0.5 µM of each primer, and 1 U of *Taq* polymerase (Vivantis Technologies, Selangor, Malaysia). PCR amplification was carried out using a Veriti™ 96-well thermal cycler (Applied Biosystems, CA, USA) under the specified cycling conditions. Distilled water and previously confirmed *bla*_CTX-M_/*bla*_CMY-2_-positive *E. coli* strains were included as negative and positive controls, respectively [14].

PCR products were analyzed by 1% agarose gel electrophoresis. Selected PCR products were purified using a DNA purification kit (RBC Bioscience, New Taipei City, Taiwan) and submitted for sequencing (First BASE Laboratories, Selangor, Malaysia). Nucleotide sequences were compared with reference sequences in the GenBank database using the Basic Local Alignment Search Tool algorithm available through the National Center for Biotechnology Information.

### Conjugation and plasmid typing assays

The transferability of *bla*_CTX-M_ and *bla*_CMY-2_ was assessed using the broth mating method with sodium azide-resistant *E. coli* J53 as the recipient strain, as previously described [15]. Donor and recipient cultures were mixed at a 1:1 ratio and incubated overnight at 37°C without shaking. Serial dilutions of the mating mixture were plated on tryptic soy agar containing cefotaxime alone, sodium azide alone, or a combination of cefotaxime and sodium azide for selection of donor, recipient, and transconjugant colonies, respectively. Positive control strains carrying *bla*_CTX-M_ or *bla*_CMY-2_ from previous studies were included [14]. All plates were incubated at 37°C overnight. Conjugation frequency was calculated as the number of transconjugants (CFU/mL) divided by the number of donor cells (CFU/mL). Transfer of resistance genes was confirmed by PCR. MICs of transconjugants were determined using the broth microdilution method according to CLSI guidelines [17]. Plasmid replicon types were identified by PCR-based replicon typing (PBRT) [21], using primers and conditions provided in Supplementary Table S1.

### Efflux pump analysis

The contribution of efflux pumps to ceftazidime resistance was evaluated by determining MICs with and without the EPI PAβN (25 µg/mL) (Sigma-Aldrich Inc., MO, USA). A previous study conducted in our laboratory demonstrated that PAβN at this concentration did not substantially affect *E. coli* growth. An MDR *P. aeruginosa* strain with known efflux pump overexpression was included as a positive control. All assays were incubated at 37°C for 18 h. A reduction of at least four-fold in ceftazidime MIC in the presence of PAβN was considered indicative of efflux pump involvement in antibiotic resistance [10].

### PFGE

Genetic relationships among 3GC-R *E. coli* isolates were examined by PFGE following established protocols [15]. Bacterial cultures were adjusted to a turbidity of 1.3–1.4 at an optical density of 625 nm, embedded in agarose plugs, digested with *Xba*I, and separated using a CHEF Mapper® XA system (Bio-Rad Laboratories, USA). Chromosome DNA from Saccharomyces cerevisiae was used as a molecular size marker. PFGE patterns were analyzed by visual inspection and interpreted according to previously described criteria [22].

### Statistical analysis

Descriptive statistics, including prevalence and AMR rates, were analyzed using Microsoft Excel. Comparisons of 3GC-R *E. coli* prevalence and antibiotic resistance rates among animal species were performed using Fisher’s exact test. Statistical analyses were conducted using Minitab version 15, and differences were considered statistically significant at p < 0.05.

## RESULTS

### Prevalence of 3GC-R *E. coli*

**Figure 1:**
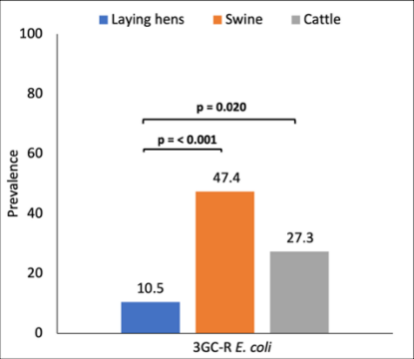
Prevalence of third-generation cephalosporin-resistant *Escherichia coli* (3GC-R *E. coli*) detected in fecal samples collected from laying hens, swine, cattle, and farm workers on a small-scale farm in northern Thailand. Bars represent the proportion of samples positive for 3GC-R *E. coli* within each host group. Statistically significant differences in prevalence among animal species were determined using Fisher’s exact test (p < 0.05).

In total, 265 fecal samples were collected from laying hens (n = 210), cattle (n = 33), swine (n = 19), and farm workers (n = 3), among which 42 samples (15.8%) yielded 3GC-R *E. coli* (Tables 1 and S2). The proportions of samples positive for 3GC-R *E. coli* were highest in swine (47.4%), followed by cattle (27.3%) and laying hens (10.5%). The prevalence of 3GC-R *E. coli* was significantly higher in swine than in laying hens (p < 0.001) (Figure 1). A similar significant difference was observed when comparing cattle and laying hens (p = 0.02). In addition, fecal samples from two of the three farm workers tested positive for 3GC-R *E. coli*.

### Antibiotic susceptibility profiles

Assessment of the antibiotic susceptibility of 3GC-R *E. coli* against 12 antibiotics showed high resistance rates to β-lactam agents. Resistance to cefotaxime and cefpodoxime was detected in 100% of isolates, whereas resistance to ceftazidime and aztreonam was observed in 73.8% and 69.0% of isolates, respectively. Resistance to amoxicillin–clavulanate and cefepime ranged from 35.7% to 38.1% (Table 2).

**Table 2:** Antibiotic resistance profiles and MDR patterns of 3GC-R *Escherichia coli* isolated from farm animals and farm workers.

Antibiotics	Number of isolates (%)				

	Laying hens (n = 22)	Swine (n = 9)	Cattle (n = 9)	Farm workers (n = 2)	Total (95% CI) (n = 42)
β-lactam					
Amoxicillin–Clavulanate	12 (54.5)	2 (22.2)	1 (11.1)	0	15 (35.7) (21.6–52.0)
Aztreonam	13 (59.0)	6 (66.7)	8 (88.9)	2 (100)	29 (69.0) (52.9–82.4)
Cefepime	7 (31.8)	4 (44.4)	3 (33.3)	2 (100)	16 (38.1) (23.6–54.4)
Cefotaxime	22 (100)	9 (100)	9 (100)	2 (100)	42 (100) (93.1; 100)
Cefpodoxime	22 (100)	9 (100)	9 (100)	2 (100)	42 (100) (93.1; 100)
Ceftazidime	19 (86.4)	5 (55.6)	5 (55.6)	2 (100)	31 (73.8) (58.0–86.1)
Imipenem	0	0	0	0	0
Non β-lactam					
Chloramphenicol	3 (13.6)	6 (66.7)	7 (77.8)	1 (50.0)	17 (40.5) (25.6–56.7)
Ciprofloxacin	2 (9.1)	3 (33.3)	0	0	5 (11.9; 4.0–25.6)
Doxycycline	6 (27.3)	7 (77.8)	7 (77.8)	1 (50.0)	21 (50.0) (34.2–65.8)
Gentamicin	7 (31.8)	6 (66.7)	7 (77.8)	1 (50.0)	21 (50.0) (34.2–65.8)
Trimethoprim + Sulfamethoxazole	9 (40.9)	6 (66.7)	5 (55.6)	2 (100)	22 (52.4) (36.4–68.0)
No. of antibiotics to which isolates are resistant					
1–3	2 (9.1)	0	0	0	2 (4.8) (0.6–16.2)
4–6	12 (54.5)	5 (55.6)	4 (44.4)	1 (50.0)	22 (52.4) (36.4–68.0)
7–9	8 (36.4)	3 (33.3)	5 (55.6)	1 (50.0)	17 (40.5) (25.6–56.7)
10	0	1 (11.1)	0	0	1 (2.4) (0.1–12.6)
Non-MDR	13 (59.1)	2 (22.2)	2 (22.2)	1 (50.0)	18 (42.9) (27.8–59.0)
MDR					
3	3 (13.6)	1 (11.1)	0	0	4 (9.5) (2.7–22.6)
4	4 (18.2)	1 (11.1)	4 (44.4)	0	9 (21.4) (10.3–36.8)
5	1 (4.5)	3 (33.3)	3 (33.3)	1 (50.0)	8 (19.0) (8.6–34.1)
6	1 (4.5)	2 (22.2)	0	0	3 (7.1) (1.5–19.5)
Total MDR	9 (40.9)	7 (77.8)	7 (77.8)	1 (50.0)	24 (57.1) (41.0–72.3)

3GC-R = Third-generation cephalosporin-resistant, MDR = Multidrug resistance (resistance to ≥3 antimicrobial classes), CI = Confidence interval. Intermediate results were interpreted as susceptible according to Clinical and Laboratory Standards Institute guidelines.

**Figure 2:**
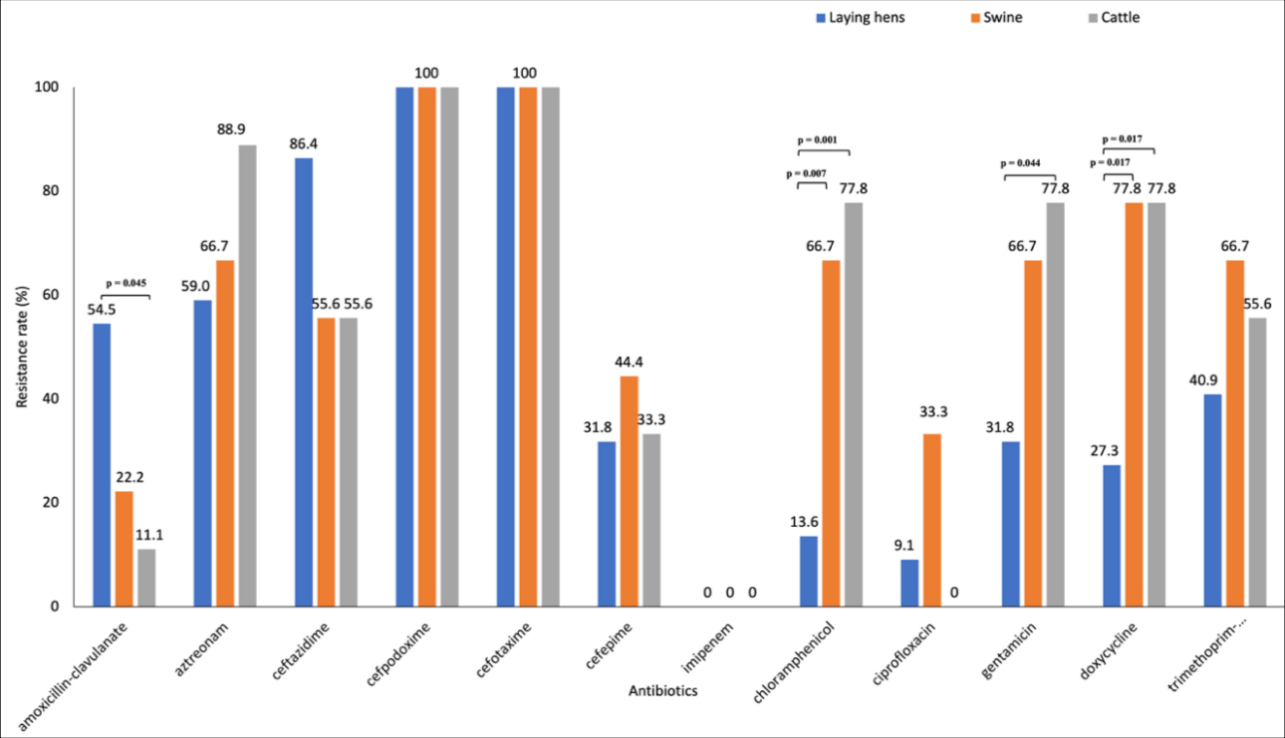
Antibiotic resistance rates of third-generation cephalosporin-resistant *Escherichia coli* isolates recovered from laying hens, swine, cattle, and farm workers. Resistance rates are expressed as percentages of isolates resistant to each tested antibiotic according to Clinical and Laboratory Standards Institute interpretive criteria. Differences in resistance profiles among host species highlight host-associated variation in antimicrobial resistance patterns.

Among non-β-lactam antibiotics, resistance to chloramphenicol, gentamicin, doxycycline, and trimethoprim–sulfamethoxazole ranged from 40.5% to 52.4%. Although resistance to ciprofloxacin was detected in 11.9% of isolates, all isolates remained susceptible to imipenem. Notably, isolates recovered from swine and cattle exhibited significantly higher resistance rates to chloramphenicol, gentamicin, and doxycycline compared with those from laying hens (Figure 2).

### MDR patterns

Most isolates (52.4%) exhibited resistance to 4–6 antibiotics, while 40.5% of isolates were resistant to 7–9 antibiotics (Table 2). A total of 24 distinct resistance profiles were identified. The most frequent resistance pattern was amoxicillin/clavulanate–ceftazidime–cefpodoxime–cefotaxime, which was detected in 14.3% of isolates (Table S3). Overall, 24 isolates (57.1%) were resistant to at least three antibiotic classes and were therefore classified as MDR. The largest proportion of isolates (21.4%) showed resistance to antibiotics belonging to four antimicrobial classes (Table 2).

### Distribution of β-lactamase genes and ESBL production

Screening for β-lactamase genes demonstrated that all 3GC-R *E. coli* isolates harbored *bla*_CTX-M_, *bla*_CMY-2_, or both (Tables 1 and S2). Twenty-nine isolates (69.0%) carried *bla*_CTX-M_ alone, including *bla*_CTX-M-55_ (n = 21), *bla*_CTX-M-14_ (n = 5), and *bla*_CTX-M_-15 (n = 3). Nine isolates (21.4%) simultaneously carried *bla*_CTX-M_ and *bla*_CMY-2_. Among the 38 *bla*_CTX-M_-positive isolates, 28 (66.7%) were confirmed as ESBL producers (Tables 1 and S2). In addition, four isolates (9.5%) carried *bla*_CMY-2_ alone.

### Transferability of resistance genes and plasmid replicon types

Conjugation experiments were performed using 23 3GC-R *E. coli* isolates as donors, including 19 *bla*_CTX-M_ positive and four *bla*_CMY-2_ positive isolates. Of these, 22 isolates—comprising 11 *bla*_CTX-M-55_, five *bla*_CTX-M-14_, two *bla*_CTX-M-15_, and four *bla*_CMY-2_, successfully transferred their resistance genes to the recipient *E. coli* J53. Conjugation frequencies ranged from 4.1 × 10^-8^ to 7.2 × 10^-2^ (Tables 3 and S4).

For transconjugants carrying either *bla*_CTX-M_ or *bla*_CMY-2_, cefotaxime MIC values were increased by up to >32-fold compared with the recipient strain (*E. coli* J53; cefotaxime MIC = 0.25 µg/mL). Among these transconjugants, 13 were successfully characterized by PBRT. Detected plasmid replicon types included IncF (n = 5), IncFIA (n = 1), IncFIB (n = 3), and IncI1-Iγ (n = 4) in transconjugants carrying *bla*_CTX-M_ and *bla*_CMY-2_ (Table 3).

**Table 3:** Transferability of *bla*_CTX-M_ and *bla*_CMY-2_ genes in 3GC-R *Escherichia coli* and associated plasmid replicon types.

*E. coli* isolates carrying *bla*_CTX-M_ or *bla*_CMY-2_	Conjugation		

	Conjugation frequency ^a^	Cefotaxime MIC (μg/mL)	Types of plasmid replicons (n)
*bla*_CTX-M-55_ (n = 11)	6.3 × 10^-8^– 7.2 × 10^-2^	0.25 >8 ^b^	IncF (n = 4) IncFIA (n = 1) Untypeable (n = 6)
*bla*_CTX-M-14_ (n = 5)	4.1 × 10^-8^– 2.6 × 10^-4^	2– >8^c^	IncI1-Iγ (n = 2) IncFIB (n = 2) IncF (n = 1)
*bla*_CTX-M-15_ (n = 2)	2.3 × 10^-7^– 5.8 × 10^-7^	>8	Untypeable (n = 2)
*bla*_CMY-2_ (n = 4)	4.2 × 10^-7^– 1.3 × 10^-3^	0.25 >8 ^b^	IncI1-Iγ (n = 2) IncFIB (n = 1) Untypeable (n = 1)

MIC = Minimum inhibitory concentration. a Conjugation frequency was calculated as the number of transconjugants divided by the number of donor cells. b One transconjugant showed a cefotaxime MIC of 0.25 µg/mL. c One transconjugant showed a cefotaxime MIC of 2 µg/mL. The MIC of cefotaxime for the recipient strain *Escherichia coli* J53 was 0.25 µg/mL.

### Contribution of efflux pumps to ceftazidime resistance

To assess the role of efflux pumps in ceftazidime resistance, 31 3GC-R *E. coli* isolates resistant to ceftazidime by disk diffusion were further examined. According to CLSI guidelines [17], *E. coli* isolates with ceftazidime MICs of 8 µg/mL and ≥16 µg/mL are categorized as intermediate and fully resistant, respectively. Accordingly, ceftazidime MIC values ranged from 16 to 128 µg/mL in 29 isolates, while two isolates exhibited MICs of 8 µg/mL (Table S2).

In the presence of PAβN, four-fold and eight-fold reductions in ceftazidime MICs were observed in 11 and one isolates, respectively (Table 4). Inhibition of efflux pump activity by PAβN in a single isolate from cattle reduced the MIC from 8 µg/mL to 2 µg/mL (Table S2), restoring susceptibility to ceftazidime.

**Table 4:** Fold reduction in ceftazidime MICs of 3GC-R Escherichia coli in the presence of an efflux pump inhibitor.

Antibiotic (No. of tested isolates)	No. (%) of isolates with indicated fold reduction in MICs after addition of PAβN				

	No change	2	4	8	16
Ceftazidime (n = 31)	6 (19.4)	13 (41.9)	11 (35.5)	1 (3.2)	0

MIC = Minimum inhibitory concentration, PAβN = Phenylalanine-arginine-β-naphthylamide. A reduction of ≥4-fold in MIC was considered indicative of efflux pump involvement.

### Genetic relatedness of 3GC-R *E. coli* isolates

**Figure 3:**
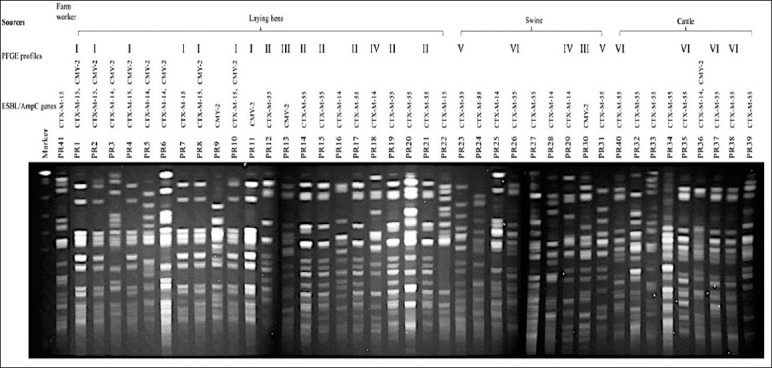
Pulsed-field gel electrophoresis (PFGE) patterns of cefotaxime-resistant *Escherichia coli* isolates obtained from laying hens, swine, cattle, and farm workers following XbaI digestion. PFGE profiles were analyzed to assess genetic relatedness among isolates from different host species. Interpretation of banding patterns was performed according to the criteria described by Tenover *et al*. [22]. Identical profiles indicate clonal relatedness, whereas distinct patterns reflect genetic diversity among isolates.

Genotypic analysis of 3GC-R *E. coli* isolates identified 23 distinct PFGE profiles (I–XXIII) among 41 isolates (Figure 3 and Table S2). One isolate from a farm worker could not be typed. Identical PFGE profiles were detected among isolates from laying hens (profiles I and II), swine (profile V), and cattle (profile VI). In addition, similarities were observed among isolates recovered from different animal species, with *E. coli* isolates from laying hens and swine sharing profiles III and IV, and isolates from swine and cattle sharing profile VI.

## DISCUSSION

### Prevalence of 3GC-R *E. coli* in small-scale livestock systems

The inappropriate use of antibiotics is a major driver of the emergence of ARB in livestock production systems. Previous studies conducted in low- and middle-income countries, particularly those focusing on small-scale farms, have reported a high prevalence of 3GC-R *E. coli* in farm animals, including chickens in Indonesia, Ecuador, and Zambia (66.1%–77.8%) [23–25], swine in Vietnam and Argentina (47.8%–89.0%) [26, 27], and cattle in Tanzania and Argentina (34.8%–95.0%) [26, 28]. Similarly, a recent study from small- and middle-scale farms in Peru reported ESBL-producing *E. coli* prevalences of 75.4% in dairy cows, 61.3% in pigs, and 34.1% in laying hens [29].

Overall, 3GC-R *E. coli* was detected in 15.8% of fecal samples collected from farm animals and workers (Table 1). This prevalence is lower than that reported in previous studies of food-producing animals raised on small-scale farms in Thailand (27.1%–55.6%) [15,16]. Differences in detection rates may be attributed to variations in study location, sample collection strategies, and isolation methodologies, all of which can influence prevalence estimates.

### Species-specific prevalence and human carriage

The highest prevalence of 3GC-R *E. coli* was observed in swine (47.4%), followed by cattle (27.3%) and laying hens (10.5%) (Table 1). This distribution is consistent with reports from other countries, including Laos, Peru, and Argentina [26, 29, 30]. The higher prevalence observed in swine may be explained by the fact that pig production systems worldwide are associated with greater antimicrobial use than other livestock sectors, leading to increased antibiotic exposure and selective pressure for resistant strains [31].

In addition to livestock, 3GC-R *E. coli* was detected in the intestinal tracts of two of the three farm workers. This finding is consistent with previous reports demonstrating a high rate of 3GC-R *E. coli* carriage among apparently healthy individuals in this region [15], underscoring the potential occupational exposure risk in small-scale farming environments.

### AMR profiles and MDR

Antimicrobial susceptibility testing revealed concerning resistance patterns. At least half of the isolates were resistant to third-generation cephalosporins (cefpodoxime and ceftazidime), aztreonam, doxycycline, gentamicin, and trimethoprim–sulfamethoxazole (Table 2), all of which are considered critically important antibiotics for the treatment of bacterial infections. Moreover, 57.1% of isolates were classified as MDR, indicating a substantial risk of treatment failure should infections occur.

### Distribution of ESBL and pAmpC genes

The presence of ESBL-producing *E. coli* in livestock represents a significant public health concern, particularly in Africa and Asia [32]. Among the 42 3GC-R *E. coli* isolates examined in this study, *bla*_CTX-M_ was predominantly detected either alone (n = 29) or in combination with *bla*_CMY-2_ (n = 9), while ESBL production was phenotypically confirmed in 28 isolates (Table 1). The absence of ESBL activity in ten *bla*_CTX-M_ positive isolates may be attributed to low-level gene expression [33]. In addition, four isolates carried *bla*_CMY-2_ alone.

The detection of *bla*_CTX-M-14_, *bla*_CTX-M-15_, *bla*_CTX-M-55_, and *bla*_CMY-2_ in farm animals aligns with findings from multiple regions worldwide [34–36], as these enzymes represent the most prevalent ESBL and plasmid-mediated AmpC β-lactamases globally [4, 37].

### Public health relevance of pAmpC-positive *E. coli* in livestock

In Thailand, pAmpC-producing *E. coli* have been frequently reported in human clinical samples [37,38], whereas reports from farm animals remain limited. Only a small number of *bla*_CMY-2_ positive *E. coli* isolates have previously been identified in domestic and wild animals, including pigs [38]. The presence of *bla*_CMY-2_ alone or in combination with *bla*_CTX-M_ in farm animals in the present study (Table 1) suggests that livestock may serve as an underrecognized reservoir for pAmpC-positive 3GC-R *E. coli* in this region.

### Horizontal transfer of resistance genes and plasmid dissemination

Conjugation experiments demonstrated that *bla*_CTX-M_ and *bla*_CMY-2_ genes carried by 3GC-R *E. coli* isolates were transferable, resulting in cefotaxime MIC increases of up to >32-fold in transconjugants. These genes were associated with IncF and IncI1-Iγ plasmids, respectively (Table 2), both of which are well known for their capacity to disseminate among Enterobacterales.

IncF plasmids are the most frequently reported plasmids in *E. coli* and commonly harbor multiple *bla*_CTX-M_ variants. Previous studies have shown that conjugative transfer of *bla*_CTX-M_ is predominantly mediated by IncF plasmids [39], consistent with findings from backyard poultry in this region [15]. Similarly, two *bla*_CMY-2_ genes in this study were located on IncI1-Iγ plasmids, which are narrow-host-range conjugative plasmids frequently detected in food animal–associated Enterobacterales [40]. On these plasmids, *bla*_CMY-2_ is often embedded within insertion sequences, particularly IS*Ecp1*, facilitating gene mobilization.

The detection of transferable *bla*_CMY-2_ on IncI1-Iγ plasmids in farm animal isolates provides new evidence of regional plasmid dissemination potential. These plasmids often carry additional resistance determinants, including those conferring resistance to fluoroquinolones and aminoglycosides, contributing to the multidrug-resistant phenotype commonly observed in 3GC-R *E. coli* [41]. The presence of such transferable resistance genes in rural farming environments represents a significant public health concern due to frequent human–animal–environment interactions.

### Role of efflux pumps in ceftazidime resistance

Efflux pump overexpression is an important contributor to resistance against multiple antibiotic classes. In this study, the contribution of efflux pumps to ceftazidime resistance was assessed using PAβN, a well-characterized inhibitor of RND efflux pumps. PAβN has been shown to reduce MICs of third-generation cephalosporins and carbapenems in several Gram-negative bacteria, including *E. coli* [42], *Klebsiella pneumoniae* [11], and *P. aeruginosa* [10].

A ≥4-fold reduction in ceftazidime MICs in the presence of PAβN was observed in 38.7% of the isolates (Table 4), indicating a contributory role of RND efflux pumps in ceftazidime resistance. Complete restoration of susceptibility in one isolate further supports efflux-mediated resistance. However, among the 12 isolates showing efflux pump overexpression, only one exhibited an MDR phenotype (Table S2), consistent with previous reports suggesting that efflux pump overexpression alone does not necessarily confer MDR. Instead, efflux-mediated resistance may progress to MDR following additional mutational events [43]. A minor (two-fold) reduction in MICs observed in 41.9% of isolates further suggests partial efflux involvement.

### Genetic relatedness and transmission dynamics

Transmission of ARB between livestock and farm workers has been documented in Japan, Vietnam, and Thailand [15, 27, 36]. Although high genetic diversity was observed among 3GC-R *E. coli* isolates in the present study, identical PFGE profiles were detected within and between animal species (Figure 3, Table S1), indicating that clonal dissemination can occur among animals raised in close proximity. Cross-species sharing of identical PFGE patterns between laying hens and swine and between swine and cattle supports local micro-ecological transmission rather than purely vertical spread.

Despite frequent interactions between animals and workers, clonal transmission between livestock and farm workers was not observed. Given the lack of direct contact among animal species, environmental dissemination via drainage water or manure is a plausible transmission route [44]. The absence of direct animal-to-human clonal spread in this study contrasts with previous reports [15, 36] and suggests that horizontal gene transfer via highly transferable plasmids carrying *bla*_CTX-M_ and *bla*_CMY-2_ (Table 3) may play a more dominant role in resistance dissemination.

### Implications for One Health surveillance and policy

The WHO and other international agencies have developed global strategies to mitigate AMR. Thailand has implemented two National Action Plans on AMR (NAP-AMR 2017–2021 and 2023–2027) [45, 46], which emphasize reducing AMR-related morbidity and mortality across human, animal, and environmental sectors. Surveillance of AMR bacteria, including 3GC-R *E. coli*, within a One Health framework is a core strategy of both plans.

Small-scale family-owned farms dominate livestock production in Thailand and are essential to the national economy. However, close human–animal contact in these systems increases zoonotic risk. The findings of this study strongly support the NAP-AMR strategy advocating expanded One Health surveillance of 3GC-R *E. coli* across humans, animals, and the environment in smallholder farming systems.

### Study limitations and future perspectives

This study has several limitations. Sampling was limited to a single farm with a relatively small sample size. Environmental samples, including feed, manure, water, and surrounding soil, were not collected, which may have provided further insight into transmission pathways. Longitudinal sampling was not performed, preventing assessment of temporal trends in 3GC-R *E. coli* prevalence. In addition, data on antibiotic usage within the farm were unavailable, limiting the ability to identify selective pressures driving resistance emergence.

Future studies should incorporate whole-genome sequencing to characterize mobile genetic elements and integrons associated with *bla* genes. Expanded genomic surveillance across humans, animals, and environmental sources on multiple smallholder farms is warranted. Strengthening biosecurity practices and maintaining antibiotic usage records at the farm-level are critical steps toward controlling the emergence and dissemination of ARB.

## CONCLUSION

This study demonstrated that 3GC-R *E. coli* are present in a small-scale, multi-species farming system in lower northern Thailand. Overall, 3GC-R *E. coli* were detected in 15.8% of fecal samples collected from laying hens, swine, cattle, and farm workers, with the highest prevalence observed in swine, followed by cattle and laying hens. A substantial proportion of isolates exhibited MDR, with more than half classified as MDR. Molecular analyses revealed that resistance was predominantly mediated by *bla*_CTX-M_ (notably *bla*_CTX-M-55_, *bla*_CTX-M-14_, and *bla*_CTX-M-15_) alone or in combination with *bla*_CMY-2_. Importantly, these resistance determinants were located on transferable IncF and IncI1-Iγ plasmids, resulting in markedly increased cefotaxime MICs in transconjugants. In addition to enzymatic mechanisms, efflux pump activity contributed to ceftazidime resistance in a subset of isolates, highlighting the multifactorial nature of resistance. Although high genetic diversity was observed, identical PFGE profiles among different animal species indicated local clonal dissemination within the farm environment.

The detection of transferable ESBL- and pAmpC-producing *E. coli* in small-scale livestock systems has important implications for animal health, food safety, and public health. The presence of mobile resistance genes on highly transmissible plasmids underscores the risk of rapid horizontal dissemination within farm ecosystems and potentially beyond the farm boundary. Smallholder farms, where animals and humans live in close proximity and biosecurity measures are often inconsistently applied, may represent critical yet under-surveilled reservoirs for AMR. These findings support the urgent need for strengthened antimicrobial stewardship, improved farm-level biosecurity, and integrated One Health surveillance that includes humans, animals, and the environment.

A major strength of this study lies in its integrated One Health approach, combining phenotypic resistance profiling, molecular characterization of β-lactamase genes, plasmid transferability assessment, efflux pump analysis, and genotypic relatedness within a single small-scale farm setting. The inclusion of multiple livestock species and farm workers allowed a comprehensive evaluation of resistance dynamics across host interfaces. Furthermore, the demonstration of both clonal spread and plasmid-mediated horizontal gene transfer provides valuable insight into the mechanisms driving the persistence and dissemination of 3GC-R *E. coli* in rural farming systems.

In conclusion, small-scale farms in Thailand can serve as reservoirs of multidrug-resistant, 3GC-R *E. coli* carrying highly transferable resistance determinants. The coexistence of enzymatic resistance, efflux pump activity, and mobile plasmids highlights the complexity of AMR ecology in livestock production systems. Expansion of One Health–based surveillance, strict implementation of biosecurity measures, and improved documentation and regulation of antibiotic use at the farm-level are essential to mitigate the further spread of 3GC-R *E. coli* within rural communities and beyond.

## DATA AVAILABILITY

The supplementary and sequencing data can be made available from the corresponding author upon request.

## AUTHORS’ CONTRIBUTIONS

UT: Methodology, fieldwork, experiment, data collection and curation, and statistical analysis and interpretation. PRN: Conceptualization and supervision, methodology, data analysis and interpretation, and original draft writing, reviewing, and editing. Both authors have read and approved the final manuscript.
